# Dexamethasone-associated metabolic effects in male mice are partially caused by depletion of endogenous corticosterone

**DOI:** 10.3389/fendo.2022.960279

**Published:** 2022-08-10

**Authors:** Lisa L. Koorneef, Merel van der Meulen, Sander Kooijman, Elena Sánchez-López, Jari F. Scheerstra, Maaike C. Voorhoeve, Ajith N. Nadamuni Ramesh, Patrick C. N. Rensen, Martin Giera, Jan Kroon, Onno C. Meijer

**Affiliations:** ^1^ Department of Medicine, Division of Endocrinology, Leiden University Medical Center, Leiden, Netherlands; ^2^ Einthoven Laboratory for Experimental Vascular Medicine, Leiden University Medical Center, Leiden, Netherlands; ^3^ Center for Proteomics and Metabolomics, Leiden University Medical Center, Leiden, Netherlands

**Keywords:** Aldosterone, corticosterone, dexamethasone, eplerenone, glucocorticoid receptor, mineralocorticoid receptor, GR-MR crosstalk, hypothalamus-pituitary-adrenal axis

## Abstract

Synthetic glucocorticoids are clinically used to treat auto-immune and inflammatory disease. Despite the high efficacy, glucocorticoid treatments causes side effects such as obesity and insulin resistance in many patients. *Via* their pharmacological target, the glucocorticoid receptor (GR), glucocorticoids suppress endogenous glucocorticoid secretion. Endogenous, but not synthetic, glucocorticoids activate the mineralocorticoid receptor (MR) and side effects of synthetic glucocorticoids may thus not only result from GR hyperactivation but also from MR hypoactivation. Here, we tested the hypothesis that reactivation of MR with corticosterone add-on treatment can attenuate the metabolic effects of the synthetic glucocorticoid dexamethasone. Male 8-week-old C57Bl/6J mice received a high-fat diet supplemented with dexamethasone or vehicle, and were subcutaneously implanted with low-dose corticosterone- or vehicle-containing pellets. Dexamethasone strongly reduced body weight and fat mass gain, while corticosterone add-on partially normalized this. Dexamethasone-induced hyperglycemia and hyperinsulinemia were exacerbated by corticosterone add-on, which was prevented by MR antagonism. In subcutaneous white adipose tissue, corticosterone add-on prevented the dexamethasone-induced expression of intracellular lipolysis genes. In brown adipose tissue, dexamethasone also upregulated gene expression of brown adipose tissue identity markers, lipid transporters and lipolysis enzymes, which was prevented by corticosterone add-on. In conclusion, corticosterone add-on treatment prevents several, while exacerbating other metabolic effects of dexamethasone. While the exact role of MR remains elusive, this study suggests that corticosterone suppression by dexamethasone contributes to its effects in mice.

## Introduction

Glucocorticoids (GCs) coordinate a wide range of metabolic processes to support the body’s requirements during normal physiology and during stress. GC secretion is under control of the hypothalamus-pituitary-adrenal (HPA)-axis, which upon activation releases corticotropin releasing factor (CRF) from the hypothalamus to stimulate pituitary adrenocorticotropic hormone (ACTH) secretion, which eventually leads to GC release by the adrenal gland (1). GCs, such as corticosterone in mice, bind to the high-affinity mineralocorticoid receptor (MR), and the lower affinity glucocorticoid receptor (GR). Given the 10-fold difference in receptor affinities, the majority of MRs are already occupied under basal, non-stressed conditions (2). Since the GR is only activated at higher GC levels, it regulates distinct aspects of the stress response and mediates negative feedback to suppress endogenous GC production (1, 2).

Dexamethasone is a synthetic GC that is used in the clinic to treat inflammatory and auto-immune diseases. While dexamethasone is relatively well-tolerated at low dosages, high-doses cause side effects such as obesity, insulin resistance, muscle atrophy and neuropsychological disturbances in more than 50% of the patients (3–6). This symptomatology is similar to that observed in Cushing’s patients with hypercortisolemia (7). In rodents, dexamethasone and corticosterone both cause insulin resistance and muscle atrophy (8–10). Corticosterone induces adiposity, but the effect of dexamethasone on body composition is not as clear, as both loss and gain of total fat mass have been reported (11–13).

Dexamethasone has a higher affinity for GR than for the MR (14), which leads to a much higher stimulation of GR *in vivo* (15). Since even moderate doses of dexamethasone can completely suppress endogenous GC production, it has been postulated that part of the (central) side effects of dexamethasone may result from MR hypoactivation rather than GR hyperactivation (16, 17). Various studies have reported beneficial effects of corticosterone or cortisol add-on treatment on mood, memory and sleep quality in humans and rodents treated with high-dose dexamethasone (18–20). In this study we evaluated the hypothesis that reactivation of MR *via* low-dose corticosterone add-on can prevent dexamethasone-induced metabolic side effects, by performing studies in male C57Bl/6J mice.

## Materials and methods

### Animals

All studies involving animals were approved by the institutional ethics committee on animal care and experimentation at the Leiden University Medical Center. Throughout all experiments, body weight, food intake and body composition (EchoMRI-100-analyzer; EchoMRI, Frankfurt, Germany) were monitored (twice) weekly.

To investigate the effects of corticosterone add-on during dexamethasone treatment, we used 8-week-old male C57Bl/6J mice (Charles River, Sulzfeld, Germany) that were housed in clear plastic cages within light-tight cabinets (21) with *ad libitum* access to food and water. For practical reasons, mice were adapted to a new 12:12 h light-dark cycle with lights on at 11:00 AM (Zeitgeber Time (ZT) 0) two weeks prior to the experiment. Mice received fructose water [10% D-(-)-fructose, Sigma-Aldrich, Zwijndrecht, the Netherlands, (22)] and a high-fat diet (HFD, 60%, Research Diets, New Brunswick, NJ), with or without dexamethasone (8.88 mg/kg diet, resulting in an effective dose of approx. 1 mg/kg/day, Sigma-Aldrich) for 23 days. In addition, mice were subcutaneously implanted with pellets containing vehicle (100 mg cholesterol, PanReac AppliChem, Meppel, the Netherlands), low-dose corticosterone (5 mg corticosterone (Sigma-Aldrich), 95 mg cholesterol) or high-dose corticosterone (20 mg corticosterone, 80 mg cholesterol; n=8 per group). Slow-release pellets were implanted while mice were under isoflurane anesthesia and buprenorphine analgesia (0.03 mg/kg, Reckitt Benckiser Healthcare, Hull, 95 United Kingdom), and were replaced after two weeks. At day 0-7, mice were housed in metabolic cages (Promethion line, Sable Systems, Las Vegas, USA) for indirect calorimetry measurements. At day 8, blood was collected at ZT1 and ZT11 within 2 min after a tail incision, i.e., before corticosterone levels start to rise due to the sampling procedure, for corticosterone measurements using a HS EIA kit (Immunodiagnostic systems, Boldon Business Park, UK). At day 9, mice were fasted for 4 h and an oral lipid tolerance test was performed at ZT1. At day 23, mice were fasted for 6 h and at ZT1, tail blood was collected for glucose and insulin measurements (using the appropriate kits of Instruchemie, Delfzijl, the Netherlands and Crystal Chem, Zaandam, Netherlands), after which uptake of triglyceride-derived fatty acids and deoxyglucose by various metabolic organs was determined (see below for details). Mice were killed by CO_2_ inhalation and perfused with ice-cold PBS for 5 min, after which tissues were isolated and stored until further processing.

In a separate cohort, we combined dexamethasone and add-on treatment with the selective MR agonist aldosterone. 8-week-old male C57Bl/6J mice were housed in individually ventilated cages with a 12:12 light-dark cycle (lights on at 07:00 AM, ZT 0) and *ad libitum* access to food and water. Mice received fructose water and a HFD mixed with vehicle or dexamethasone (1 mg/kg/day). In addition, mice were subcutaneously implanted with pellets containing vehicle (100 mg cholesterol) or aldosterone (100 mg cholesterol with 0.3 mg aldosterone; Sigma-Aldrich) for 10 days (n=7 per group). At day 9, blood was collected within 2 min after a tail incision for determination of plasma corticosterone levels at ZT1 and ZT11. At day 10, mice were fasted for 6 h and at ZT1, blood was collected for measurement of plasma glucose, insulin, free fatty acid and triglyceride levels (Roche, Almere, the Netherlands, Instruchemie, Crystal Chem). Mice were killed by CO_2_ inhalation and perfused with ice-cold PBS for 5 min, after which tissues were isolated and stored until further processing.

To evaluate the MR as a mediator of corticosterone effects, we treated mice with the MR antagonist eplerenone. 8-week-old male C57Bl/6J mice were housed in individually ventilated cages with a 12:12 light-dark cycle (lights on at 7:00 AM, ZT 0) and *ad libitum* access to food and water. Mice received fructose water and a HFD mixed with vehicle, dexamethasone (1 mg/kg/day), and dexamethasone with eplerenone (1066 mg/kg diet, resulting in an effective dose of approx. 120 mg/kg/day) for 23 days. In addition, mice were subcutaneously implanted with pellets containing vehicle (100 mg cholesterol) or low-dose corticosterone (5 mg corticosterone, 95 mg cholesterol) which were replaced after two weeks. Group sizes were n=6 for all dexamethasone groups and n=3 for the vehicle group. At day=10, mice were fasted for 6 h after which an oral glucose tolerance test was performed at ZT1. At day=21, tail blood was collected at ZT1 and ZT11 for corticosterone measurements. At day=23, mice were fasted for 6 h, tail blood was collected for white blood cell counting after which mice were killed by CO_2_ inhalation and perfused with ice-cold PBS for 5 min. Tissues were isolated and stored until further processing.

### Oral lipid tolerance test

To measure the post-prandial lipid response, a baseline 6 h fasted blood sample was collected in paraoxon-coated capillaries *via* a tail incision, after which mice received an oral olive oil bolus (200 µl, Carbonell, Cordoba, Spain). Additional blood samples were collected after 1, 2, 4 and 6 h and plasma triglyceride and free fatty acid levels were determined using commercially available kits (Roche).

### Plasma clearance and organ uptake of triglyceride-derived fatty acids

Triglyceride-rich lipoprotein (TRL)-like particles, 80 nm in diameter and labeled with glycerol tri[^3^H]oleate, were prepared as previously described (23). 6 h fasted mice were intravenously injected with 200 µL emulsion containing 1 mg triglycerides and a tracer amount of [^14^C]-deoxyglucose. Additional blood samples were collected after 2, 5, 10 and 15 min to evaluate the plasma decay of the radiolabels. After collection of various metabolic active tissues, [^3^H]- and [^14^C]-activities were determined using liquid scintillation counting.

### Oral glucose tolerance test

To measure glucose tolerance, a 6 h fasted blood sample was collected in paraoxon-coated capillaries *via* a tail incision. Next, a glucose bolus was administered orally (1 g/kg), and after 5, 15, 30, 60, and 120 min additional blood samples were collected for plasma glucose measurement using the Accu-Chek Aviva (Roche).

### White blood cell counting

Blood concentrations of the major leukocyte classes, i.e. lymphocytes, monocytes and eosinophils, were determined in whole blood using an automatic veterinary hematology analysis (Sysmex XT-2000iV; Goffin Meyvis, Etten-Leur, the Netherlands).

### Liquid chromatography-mass spectrometry analysis

Frozen liver and white adipose tissue (WAT) tissue samples were weighted and homogenized in chilled LC-MS-grade water or 2-isopropanol respectively (final concentration: 200 mg/mL) with the Bullet Blender™ 24 (Next Advance Inc., NY, USA). For steroid extraction, 10 µL of the internal standards (IS,100 ng/mL of cortisol-d_4_ (Sigma-Aldrich), testosterone-d_3_ (Sigma-Aldrich) and progesterone-d_9_ (Sigma-Aldrich) in methanol) and 190 µL ice-cold LC-MS grade methanol were added to 25 µL of tissue homogenate. Samples were vortexed, centrifuged and supernatants were collected, after which extraction steps were repeated and the extracts combined. Samples were dried under a gentle stream of N_2_ (0.8 L/min, 40°C) and stored at -80°C until reconstitution.

On the day of analysis, samples were reconstituted in LC-MS grade methanol, vortexed and sonicated for 1 min. Next, additional LC-MS grade water was added, after which samples were vortexed, centrifuged and the supernatant collected into micro-vial inserts for placement in the autosampler for LC-MS analysis. Sample type specific quality control (QC) samples were prepared by combining 6 µL from each individual sample. For steroid quantification, eight solutions containing a mixture of the authentic synthetic standards (cortisol (Sigma-Aldrich), cortisone (Sigma-Aldrich), corticosterone (Sigma-Aldrich), 11-dehydrocorticosterone (Biochemicals), dexamethasone (Sigma-Aldrich), testosterone (Sigma-Aldrich), dihydrotestosterone (Sigma-Aldrich) and progesterone (Sigma-Aldrich)) were prepared in a concentration range from 0.01 to 50 ng/mL for each steroid. All solutions included 10 µL of the IS mix (100 ng/mL in methanol), and had a final composition of methanol:water of 40:60 (v/v).

Steroid analysis was performed using a QTrap 6500+ mass spectrometer in positive electrospray (ESI) mode (Sciex, Nieuwerkerk aan den Ijssel, the Netherlands) coupled to an HPLC system employing a DGU-403 degassing unit, LC-40D pumps, a SIL-40C auto sampler, and a CTO-40S column oven (Shimadzu, ‘s-Hertogenbosch, the Netherlands). The chromatographic separation took place in a Kinetex 50 x 2.1 mm, 1.7 µm C18 column with a C18 pre-column (Phenomenex, Utrecht, the Netherlands), kept at 50°C. LC-MS water and methanol both containing 0.1% formic acid were used as mobile phase A and B, respectively. The gradient, with a column flow rate of 400 µL/min, was as follows: 40% B at 0 min, 48.8% B at 3.5 min, 60.0% B at 4.0 min, 85% B at 6.5 min, 100% B at 6.6 min, 100% B at 8.6 min, and 40% B at 9.5 min. The injection volume was 10 µL. The MRM transitions used to identify the different steroids are included in **Supplementary Table S1**. Other MS parameters include a curtain gas of 20 psi, ion source gas #1 and #2 of 40 and 20 psi, respectively, a “medium” collision gas, an ionspray voltage of 5500 V, a source temperature of 350°C, an ion source gas of 40 V, and an entrance potential of 10 V. Samples were analyzed in a randomized fashion. QCs were injected every six samples to evaluate consistency along the whole analytical run.

### RNA isolation, cDNA synthesis and real-time quantitative PCR

Total RNA was isolated from snap frozen tissues using TriPure isolation reagent (Roche). After reverse transcription to cDNA (Promega, Leiden, the Netherlands), RT-qPCR was performed using IQ SYBR-Green supermix (Promega) and MyIQ thermal cycler (Bio-RAD CFX96, Veenendaal, Netherlands). Primers were tested for high efficiency (90%-110%) and for amplification of a single PCR product. All primer sequences are listed in **Supplementary Table S2**, and fold change expression was calculated using the 2*^-ΔΔCT^* method using ribosomal protein S18 as housekeeping gene in all tissues, except for the hippocampal data from the first corticosterone add-on experiment where beta-2 microglobulin was used.

### Western blot

Proteins were isolated from snap frozen brown adipose tissue (BAT) by lysing tissues with RIPA buffer supplemented with protease inhibitors, after which the protein lysate was collected. Western blot was performed loading 4 µL of 0.2 µg/mL lysate on the Wes apparatus (ProteinSimple, Wiesbaden, Germany), according to the manufacturer’s protocol. The following primary and secondary antibodies were used: UCP1 (Sigma-Aldrich, U6382, 1:20), GAPDH (Santa Cruz Biotechnology, sc25778, 1:50), HRP anti-rabbit (ProteinSimple, DM001) and HRP anti-sheep (Abcam, Amsterdam, the Netherlands, ab6900, 1:100).

### Histology

Interscapular BAT (iBAT), gonadal WAT (gWAT), subcutaneous WAT (sWAT) and liver tissue were fixed in 4% paraformaldehyde for 24 h and stored in 70% ethanol until further processing. Tissues were dehydrated, embedded into paraffin, and cut into 5 µm sections. Paraffin-waxed tissues were dewaxed and dehydrated before staining with Mayers Haematoxylin (Merck, Netherlands) and eosin (Sigma-Aldrich). Adipocyte size and lipid content was quantified using ImageJ software [National Institutes of Health, Bethesda, MD (24)]. The scoring system for hepatic steatosis and characteristics was adapted from (25).

### Statistics

**Figure 1:**
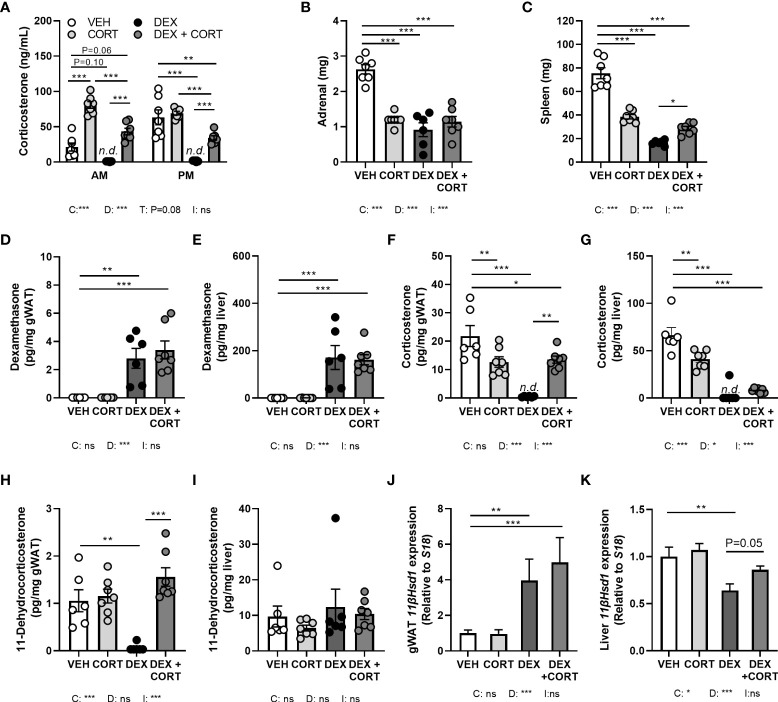
Dexamethasone depletes corticosterone levels in blood and glucocorticoid target tissues, while corticosterone add-on partially restores this. Mice received a high-fat diet mixed with dexamethasone (DEX) or vehicle (VEH) and were subcutaneously implanted with low-dose (5 mg) corticosterone (CORT) or vehicle pellets for 3.5 weeks. **(A)** After DEX, plasma CORT levels were not detected (n.d.) at AM (ZT1) and PM (ZT11) of the light phase at day 8. **(B, C)** DEX strongly reduced adrenal and spleen weight. **(D, E)** Using LC-MS analysis, DEX levels were 100-fold higher in liver than in gonadal white adipose tissue (gWAT). **(F, G)** In gWAT and liver, DEX strongly suppressed CORT levels, which were (partially) restored by DEX + CORT. **(H, I)** DEX suppressed 11-dehydrocorticosterone levels in gWAT but not in liver. **(J, K)** *11βHsd1* expression was upregulated by DEX and DEX + CORT in gWAT and downregulated by DEX in liver. Statistical significance was calculated using 2-way ANOVA analysis in **(B-K)**, and 3-way ANOVA analysis in **(A)** Depicted below the graphs are the P-values of the ANOVA tests for either time (T), CORT (C), DEX (D) or the interaction between CORT and DEX (I). *P < 0.05, ** P < 0.01, ***P < 0.001. 'ns' means 'not significant'. n.d. means 'not detected'.

**Figure 2:**
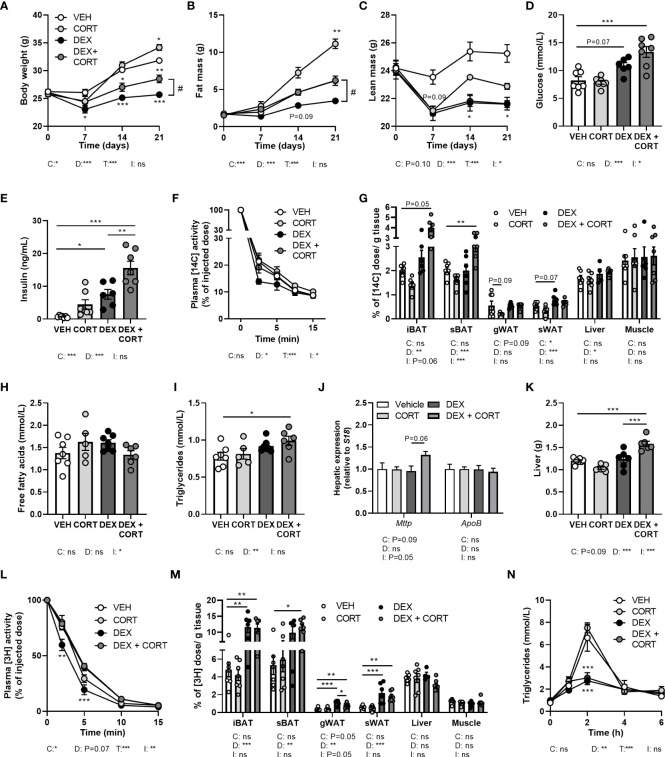
Corticosterone add-on treatment reverses dexamethasone-induced fat mass loss. Mice received a high-fat diet mixed with dexamethasone (DEX) or vehicle (VEH) and were subcutaneously implanted with low-dose (5 mg) corticosterone (CORT) or vehicle pellets for 3.5 weeks. **(A–C)** DEX decreased body weight, fat and lean mass, while DEX + CORT prevented the effects on body weight and fat mass. Asterisks show results of *post-hoc* comparisons with VEH, hash marks of *post-hoc* comparisons with the DEX. **(D, E)** DEX increased 6 h-fasted glucose and insulin levels after 23 days, while DEX + CORT aggravated this effect. **(F)** At AM, DEX induced a more rapid plasma clearance of intravenously injected [^14^C]-deoxyglucose, which was prevented by DEX + CORT. **(G)** DEX + CORT increased [^14^C]-deoxyglucose uptake in interscapular brown adipose tissue (BAT). **(H)** 4 h-fasted plasma free fatty acid levels were unaffected in all treatment groups. **(I, J)** DEX + CORT increased 4 h-fasted plasma triglyceride levels and hepatic expression of *Mttp* but not *Apob*. **(K)** DEX + CORT increased liver weight. **(L)** DEX induced a more rapid plasma clearance of intravenously injected triglyceride-rich lipoprotein-like particles containing glycerol tri[^3^H]oleate. **(M)** DEX and DEX + CORT similarly increased the uptake [^3^H]oleate in BAT and white adipose tissue (WAT) depots. **(N)** Upon an oral lipid bolus, DEX strongly reduced peak plasma triglyceride levels. Statistical significance was calculated using 2-way ANOVA analysis in **(D, E, G–K, M)**; with 3-way ANOVA analysis in **(F, L)**; with linear mixed models in **(A–C, N)**. Depicted below the graphs are the P-values of the ANOVA tests for either time (T), CORT (C), DEX (D) or the interaction between CORT and DEX (I). *P < 0.05, ** P < 0.01, ***P < 0.001. 'ns' means 'not significant'.

**Figure 3:**
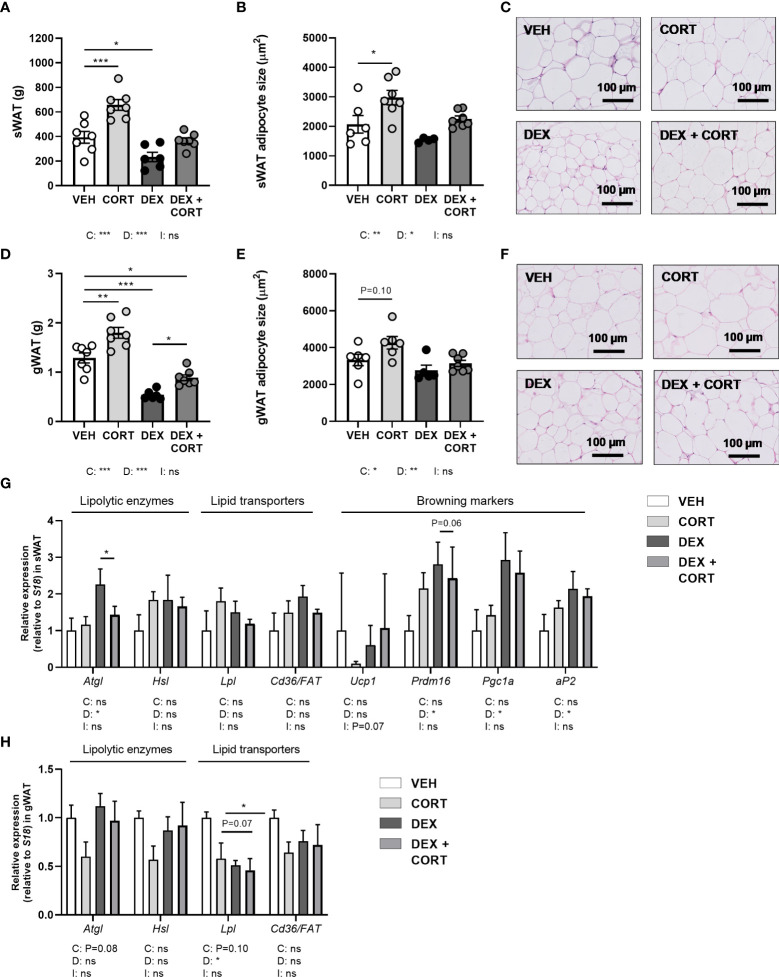
Dexamethasone stimulates expression of lipolytic enzyme *Atgl* in WAT, which is prevented by corticosterone add-on treatment. Mice received a high-fat diet mixed with dexamethasone (DEX) or vehicle (VEH) and were subcutaneously implanted with low-dose (5 mg) corticosterone (CORT) or vehicle pellets for 3.5 weeks. DEX decreased tissue weight and adipocyte size, as determined by H&E staining, in **(A–C)** subcutaneous white adipose tissue (sWAT) and in **(D–F)** gonadal white adipose tissue (gWAT). **(G)** In sWAT, DEX increased the expression of lipolytic enzyme *Atgl* and browning markers *Prdm16, Pgc1a* and *aP2.* DEX + CORT prevented the DEX-induced upregulation of *Atgl* expression. **(H)** In gWAT, DEX did not affect *Atgl, Hsl*, or *Cd36/FAT* expression, but decreased *Lpl* expression. Statistical significance was calculated using 2-way ANOVA analysis. Depicted below the graphs are the P-values of the ANOVA tests for either CORT (C) or DEX (D) treatment or the interaction between CORT and DEX (I). *P < 0.05, ** P < 0.01, ***P < 0.001. 'ns' means 'not significant'.

**Figure 4:**
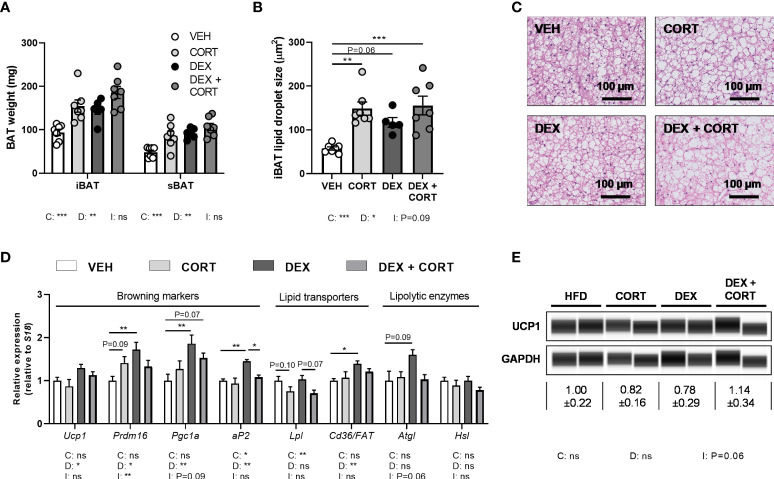
Dexamethasone increases expression of BAT activation markers genes, which is counteracted by corticosterone add-on treatment. Mice received a high-fat diet mixed with dexamethasone (DEX) or vehicle (VEH) and were subcutaneously implanted with low-dose (5 mg) corticosterone (CORT) or vehicle pellets for 3.5 weeks. **(A–C)** CORT and DEX increased the weight of interscapular brown adipose tissue (iBAT) and suprascapular brown adipose tissue (sBAT), and increased the lipid content of iBAT, as determined by H&E staining. **(D)** In iBAT, DEX increased expression of *Ucp1*, *Prdm16*, *Pgc1a*, *aP2*, *Cd36/FAT* and *Atgl*, which was (partially) reversed by CORT add-on treatment. **(E)** None of the treatments affected UCP1 protein expression in sBAT. Statistical significance was calculated using 2-way ANOVA analysis. Depicted below the graphs are the P-values of the ANOVA tests for either CORT (C), DEX (D) or the interaction between CORT and DEX (I). *P < 0.05, ** P < 0.01, ***P < 0.001. 'ns' means 'not significant'.

**Figure 5:**
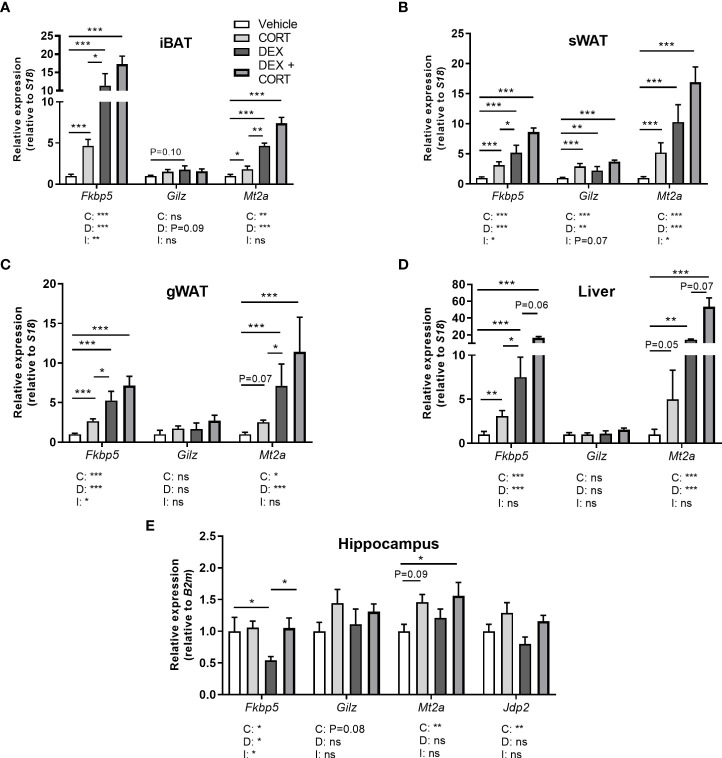
Corticosterone adds to dexamethasone-induced gene expression. Mice received a high-fat diet mixed with dexamethasone (DEX) or vehicle (VEH) and were subcutaneously implanted with low-dose (5 mg) corticosterone (CORT) or vehicle pellets for 3.5 weeks. **(A–D)** DEX increased expression of GR target genes *Fkbp5, Gilz* and *Mt2a* stronger than CORT in interscapular brown adipose tissue (iBAT), subcutaneous white adipose tissue (sWAT), gonadal white adipose tissue (gWAT) and liver, while the induction after DEX + CORT was highest. **(E)** In the hippocampus, DEX decreased *Fkbp5* expression and did not regulate *Gilz* and *Mt2a* expression. DEX + CORT normalized *Fkbp5*, and induced *Gilz*, *Mt2a* and *Jdp2* expression. Statistical significance was calculated using 2-way ANOVA analysis. Depicted below the graphs are the P-values of the ANOVA tests for either CORT (C), DEX (D) or the interaction between CORT and DEX (I). *P < 0.05, ** P < 0.01, ***P < 0.001. 'ns' means 'not significant'.

Statistical analyses were performed with GraphPad Software (version 8.1.1.330, La Jolla, CA, USA). All data are expressed as mean ± SEM. All p-values are two-tailed and p<0.05 was considered as statistically significant. Balanced data with one, two or three factors were analyzed with a one-way, two-way or three-way ANOVA respectively, and a Tukey *post-hoc* test. Figures show the *post-hoc* comparisons with the vehicle and dexamethasone-only group, with the exception of **Figure 5** and all graphs showing plasma corticosterone levels which also show comparisons with the corticosterone-only group. Below graphs showing the results of a 2-way ANOVA analysis, the statistical significance is reported of all main effects, i.e. the effect of only one of the independent variables on the dependent variable. Unbalanced data were analyzed with linear mixed models. In the first corticosterone add-on experiment, results from the low-dose and high-dose corticosterone groups were presented in separate figures (i.e., **Figures 1**–**5** (low-dose) and **Supplementary Figure S3** (high-dose). In the eplerenone experiment, the vehicle group was excluded from all statistical analyses, as its purpose was as an experimental positive control for the effects of dexamethasone, rather than part of the factorial design of the experiment. All statistical tests were performed in GraphPad Prism for Windows, version 8.1.1.

## Results

### Dexamethasone treatment depletes corticosterone levels in blood and glucocorticoid target tissues, while corticosterone add-on partially restores this

To investigate to what extent corticosterone depletion mediates the metabolic effects of dexamethasone and whether corticosterone add-on may prevent this, male C57Bl/6J mice received a HFD with or without added dexamethasone (approx. 1 mg/kg/day) in combination with low-dose corticosterone (5 mg) or vehicle pellets for 23 days. We first evaluated HPA-axis suppression by measuring corticosterone levels at the trough (AM, ZT1) and peak (PM, ZT11). Vehicle-treated mice displayed a normal circadian rhythmicity of corticosterone levels with low levels at AM and high levels at PM, while corticosterone-treated animals had constant corticosterone levels throughout the day, of which the level was comparable to that of vehicle-treated mice at PM (**Figure 1A**). Dexamethasone completely suppressed endogenous corticosterone levels (**Figure 1A**), while corticosterone add-on treatment increased plasma corticosterone levels (**Figure 1A**). Dexamethasone, corticosterone and the combination treatment all strongly reduced the weight of the GC-sensitive adrenal and spleen, as compared to vehicle-treated animals (**Figures 1B, C**). Using LC-MS analysis, dexamethasone levels were found to be 100-fold higher in liver than in gWAT independent of combined treatment with corticosterone (**Figures 1D, E**). In both gWAT and liver, corticosterone levels were strongly reduced after treatment with dexamethasone alone (**Figures 1F, G**). In gWAT and liver, corticosterone add-on increased local corticosterone levels (**Figures 1F, G**). Interestingly, dexamethasone strongly suppressed 11-dehydrocorticosterone levels in gWAT, but not in the liver (**Figures 1H, I**). Since 11-dehydrocorticosterone is used as substrate for the 11β-hydroxysteroid dehydrogenase type 1 enzyme (11-βHSD1), *11βHsd1* expression was measured in the gWAT and liver. In gWAT, *11βHsd1* expression was similarly upregulated after dexamethasone with or without corticosterone add-on, which could underlie the observed local 11-dehydrocorticosterone levels (**Figure 1J**). In the liver *11βHsd1* expression was downregulated by dexamethasone alone (**Figure 1K**). In summary, dexamethasone depleted endogenous corticosterone levels in plasma and GC-sensitive tissues WAT and liver, while corticosterone add-on could partially restore this.

### Corticosterone add-on treatment partially prevents the dexamethasone-induced loss of body weight and fat mass

To evaluate metabolic effects of dexamethasone and corticosterone add-on, we measured body weight and composition over the course of the 21-day experiment. We observed fat mass gain in HFD vehicle-treated mice, which was exacerbated by corticosterone treatment (**Figures 2A–C**) while dexamethasone prevented body weight and fat mass gain (**Figures 2A, B**). Corticosterone add-on treatment counteracted the dexamethasone-induced effects on fat mass and in part on body weight (**Figures 2A, B**). Dexamethasone reduced lean mass which could not be prevented by corticosterone add-on treatment (**Figure 2C**). Indirect calorimetry during the first week of treatment showed that dexamethasone decreased fructose water intake and locomotor activity (main effect: P<0.01), while food intake was not affected (**Supplementary Figures S1A–C**). Dexamethasone increased total and light phase respiratory quotient as well as light and dark phase energy expenditure (main effects: P<0.05), which is linked to higher carbohydrate oxidation in these groups (main effects: P<0.01), but this was not influenced by corticosterone add-on (**Supplementary Figures S1D–I**). Fat oxidation was not affected by dexamethasone and corticosterone (main effects: non-significant (ns), **Supplementary Figures S1J, K**). It should be noted that all significant effects on energy expenditure, carbohydrate and fat oxidation were only observed when expressing the data per gram lean mass and uncorrected data revealed no effects of dexamethasone or corticosterone add-on treatment (**Supplementary Figures S1L–N**). In conclusion, dexamethasone prevented HFD-induced body weight and fat mass gain, which was counteracted by corticosterone add-on treatment and these effects were not fully attributed to changes in energy expenditure, locomotion or food intake.

### Corticosterone add-on treatment aggravates the dexamethasone-induced elevation of plasma glucose and insulin levels

Given that insulin resistance is a common side effect of dexamethasone (26, 27), we determined treatment effects on glucose metabolism. Dexamethasone increased fasted glucose levels (main effect: P<0.001) and corticosterone add-on aggravated this effect (interaction term: P<0.05, **Figure 2D**). Insulin levels were increased by dexamethasone and this was also exacerbated by corticosterone add-on (**Figure 2E**). Dexamethasone led to a more rapid plasma clearance of intravenously administered [^14^C]-deoxyglucose compared to the vehicle group (main effect: P<0.05), while corticosterone add-on prevented this (interaction term: P<0.05, **Figure 2F**). Dexamethasone increased the uptake of [^14^C]-deoxyglucose in sWAT, while corticosterone add-on reduced it (main effects: P<0.05) (**Figure 2G**). Dexamethasone did not affect [^14^C]-deoxyglucose uptake in iBAT and subscapular BAT (sBAT), while corticosterone add-on increased [^14^C]-deoxyglucose uptake in sBAT (*post-hoc* tests: P<0.01, **Figure 2G**). In liver, [^14^C]-deoxyglucose uptake was slightly increased by dexamethasone (main effect: P<0.05), while we observed no effects in quadriceps muscle (**Figure 2G**). All in all, corticosterone add-on exacerbated the dexamethasone-induced hyperglycemia and hyperinsulinemia, while also increasing glucose uptake in BAT.

### Dexamethasone increases plasma triglycerides, which is exacerbated by corticosterone add-on

With respect to lipid metabolism, dexamethasone and corticosterone add-on did not alter fasted free fatty acid levels (**Figure 2H**) while fasted plasma triglyceride levels were increased after dexamethasone treatment (main effect: P<0.01) which was more pronounced after corticosterone add-on treatment (*post-hoc*: P<0.01, **Figure 2I**). The observed increase in plasma triglycerides can reflect an elevation of very-low-density-lipoproteins (VLDL) levels, and we therefore measured hepatic expression of genes involved in VLDL production, i.e., mitochondrial transfer protein (*Mttp*) and apolipoprotein B (*ApoB*). None of the treatments significantly affected *Mttp* and *ApoB* expression (**Figure 2J**). Dexamethasone alone did not alter liver weight, but dexamethasone with corticosterone add-on treatment significantly increased liver weight (*post-hoc* tests: P<0.001, **Figure 2K**). Histological examination of the liver showed that vehicle-treated HFD-fed animals had a mild to moderate microvesicular steatosis, and that corticosterone add-on additionally induced macrovesicular steatosis (**Supplementary Figures S2A–C**). Dexamethasone treatment caused hepatocellular ballooning, irrespective of corticosterone (**Supplementary Figure S2D**).

### Dexamethasone increases the uptake of triglyceride-derived fatty acids in BAT

To further determine treatment effects on lipid fluxes mice were injected with TRL-like particles labeled with glycerol tri[^3^H]oleate. Compared to the vehicle group, clearance of [^3^H]-derived activity from the circulation was more rapid after treatment with dexamethasone alone, while corticosterone add-on normalized this effect (**Figure 2L**). Dexamethasone increased the uptake of [^3^H]oleate, derived from glycerol tri[^3^H]oleate, by iBAT and sWAT (main effects: P<0.01), while corticosterone add-on did not influence this (**Figure 2M**). In gWAT, dexamethasone increased the uptake of triglyceride-derived [^3^H]oleate (main effect: P<0.01), which was partially counteracted by corticosterone add-on (*post-hoc* test: P<0.05, **Figure 2M**). Uptake of [^3^H]oleate in liver and quadriceps muscle was unaffected by all treatments (**Figure 2M**). The dexamethasone stimulation of the uptake of [^3^H]oleate and glucose in BAT suggests a higher metabolic activity of the tissue, which may influence post-prandial plasma triglyceride levels (28). To investigate this hypothesis, mice received an oral bolus of triglycerides at AM (ZT1), when BAT is least active (28). Dexamethasone strongly reduced peak plasma triglyceride levels compared to the vehicle-treated animals after triglyceride administration, but this was not further influenced by corticosterone add-on (**Figure 2N**). To conclude, the dexamethasone-increased uptake of triglyceride-derived fatty acids by BAT, together with the increased clearance of post-prandial lipid levels suggest that dexamethasone induces a higher metabolic activity of BAT, which was not affected by corticosterone add-on treatment.

### Dexamethasone reduces white adipocyte size and increases *Atgl* expression in WAT, which are all partially prevented by corticosterone add-on

In line with the decrease in total fat mass, dexamethasone decreased tissue weights of gWAT and sWAT and adipocyte size (main effects: P<0.05), while corticosterone add-on prevented this (main effects corticosterone: P<0.05, **Figures 3A–F**). Corticosterone alone increased WAT weight and adipocyte size as compared to vehicle (**Figures 3A–F**). To investigate whether the decreased WAT weight after dexamethasone was a result of enhanced lipolysis, we evaluated gene expression of the lipolysis enzymes hormone sensitive lipase (*Hsl*) and adipose triglyceride lipase (*Atgl*). Interestingly, dexamethasone increased *Atgl* expression (main effect: P<0.05), and corticosterone add-on treatment prevented this increase (*post-hoc* test: P<0.05) (**Figure 3G**). *Hsl* expression was unaffected by dexamethasone and corticosterone treatment (**Figure 3G**). As the elevated influx of triglyceride-derived fatty acids into sWAT may indicate a browning of the tissue (29), we evaluated expression of genes involved in browning and lipid transport. Dexamethasone increased the expression of the browning markers PR domain containing 16 (*Prdm16*), peroxisome proliferator-activated receptor gamma coactivator 1-alpha (*Pgc1a*) and adipocyte protein 2 (*aP2*) (main effects: P<0.05) but not uncoupling protein 1 (*Ucp1*), while corticosterone add-on treatment did not significantly affect this (**Figure 3G**). In gWAT, *Atgl, Hsl* and *Cd36/FAT* expression were unaffected by dexamethasone and corticosterone treatment (**Figure 3H**). Lipoprotein lipase (*Lpl*) expression was decreased by dexamethasone (main effect: P<0.05), but not by corticosterone (**Figure 3H**). Overall, the dexamethasone-induced decrease of lipid content in WAT may in part be related to increased lipolysis and browning. Corticosterone add-on prevented this reduction of adipocyte size, potentially by preventing the induction of *Atgl* by dexamethasone.

### Dexamethasone increases BAT activation markers, which is prevented by corticosterone add-on treatment

BAT activation might also contribute to the observed fat mass loss upon dexamethasone treatment. Since dexamethasone increased energy expenditure and influx of lipids into the BAT, we investigated additional markers of BAT activity. Dexamethasone and corticosterone increased the weight and lipid content of iBAT (main effects: P<0.05, **Figures 4A–C**). Dexamethasone treatment increased expression of BAT identity marker genes *Ucp1, Prdm16, Pgc1a* and *aP2* (main effects: P<0.05). Corticosterone add-on treatment normalized this for *Prdm16* (interaction term: P<0.01) and *Ap2* (*post-hoc* test: P<0.05, **Figure 4D**). Dexamethasone did not influence *Atgl, Hsl* and *Lpl* expression, while *Lpl* was significantly decreased by corticosterone (main effect: P<0.01, **Figure 4D**). We did not detect an effect on UCP1 protein levels by dexamethasone, corticosterone or the combination (**Figure 4E**). Together these data suggest again that dexamethasone increased BAT activity, which was counteracted by corticosterone add-on treatment.

### Corticosterone add-on enhances dexamethasone-induced GR and MR target gene expression

To evaluate effects on corticosteroid receptor activity, we investigated expression of metallothionein 2 (*Mt2a*), FK506-binding protein 51 (*Fkbp5*), glucocorticoid-induced leucine zipper (*Gilz/Tsc22d3*) and *Jun dimerization protein 2* (*Jdp2*) in iBAT, sWAT, gWAT, liver and hippocampus. *Fkbp5*, *Gilz* and *Mt2a* are known to be regulated by both GR and MR (30–32). GR (*Nr3c1*) and MR (*Nr3c2*) were expressed in iBAT, sWAT, liver and hippocampus (**Supplementary Table S3**). In iBAT, sWAT, gWAT, and liver, dexamethasone stimulated *Fkbp5* expression more strongly than corticosterone treatment and a similar pattern was observed for *Mt2a* in iBAT and gWAT (**Figures 5A–D**). Corticosterone and dexamethasone additively enhanced the expression of *Fkbp5* and *Mt2a* in liver, and of *Mt2a* in iBAT and gWAT (main effects: P<0.001; interaction term: ns, **Figures 5A–D**). A different pattern was observed in the hippocampus, where dexamethasone did not influence the expression of *Gilz* and *Mt2a* (main effects: P= ns, **Figure 5E**) but decreased *Fkbp5* expression (**Figure 5E**), which is likely related to the poor brain penetration of dexamethasone (33). Corticosterone upregulated *Mt2a* expression (main effect: P<0.01) but did not influence *Gilz* expression (**Figure 5E**). Corticosterone add-on prevented the reduction of *Fkbp5* expression by dexamethasone (*post-hoc* tests: P<0.05, **Figure 5E**). Since we hypothesized that the MR may mediate the effects of corticosterone add-on treatment, we additionally evaluated expression of putative MR-responsive target gene *Jdp2* in the hippocampus (30) (**Figure 5E**). Dexamethasone did not affect *Jdp2* expression, while corticosterone induced *Jdp2* expression (main effect: P<0.01, **Figure 5E**). Taken together, these results show that dexamethasone has distinct effects on transcriptional regulation in peripheral tissues and the brain. In the periphery, corticosterone add-on further increased GR target gene expression on top of that induced by dexamethasone.

### High-dose corticosterone causes other metabolic effects than dexamethasone

**Figure 6:**
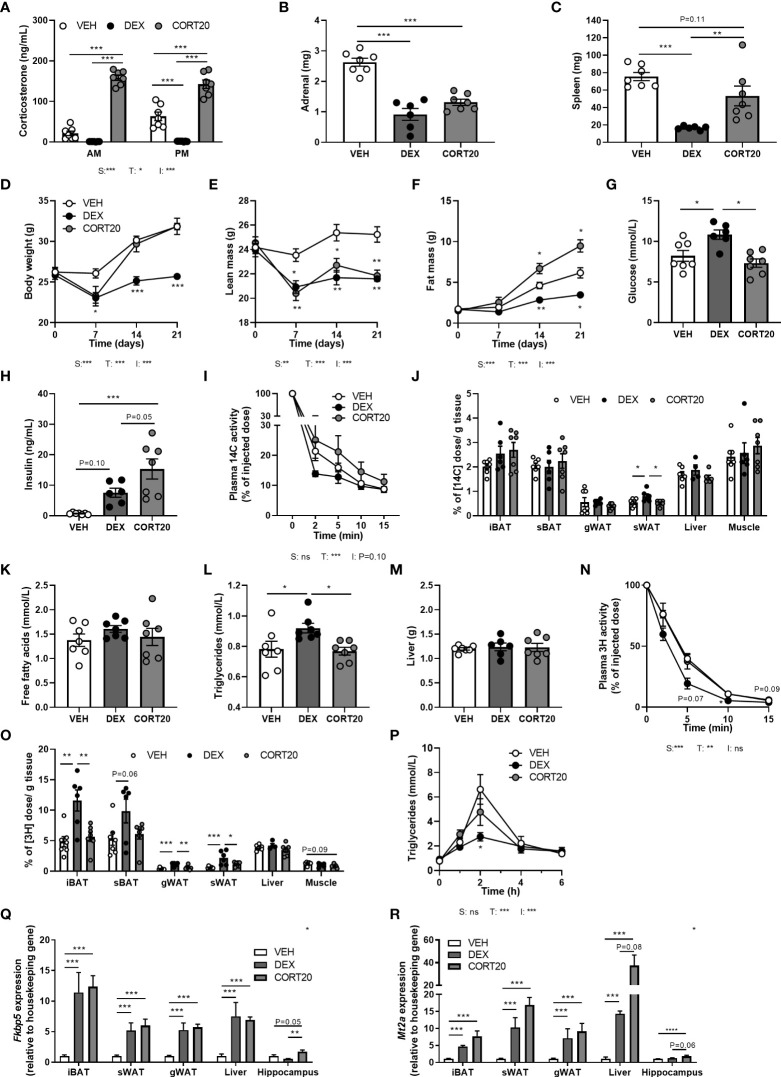
High-dose corticosterone causes different metabolic effects than dexamethasone. Mice received a high-fat diet mixed with dexamethasone (DEX) or vehicle (VEH) and were subcutaneously implanted with high-dose corticosterone (CORT20) or vehicle pellets for 3.5 weeks. The VEH and DEX groups are identical as those depicted in **Figures 1**–**5**. **(A–C)** CORT20-treated mice had strongly elevated CORT levels and decreased adrenal weight but not spleen weight. **(D–F)** CORT20 increased body weight and fat mass, but decreased lean mass. **(G, H)** CORT20 did not affect 6 h-fasted glucose levels, but increased insulin levels. **(I, J)** After 6 h-fasting at ZT1, CORT20 did not affect the plasma clearance of [^14^C]-deoxyglucose or uptake in peripheral tissues. **(K–M)** CORT20 did not influence 4 h-fasted plasma free fatty acids, triglycerides or liver weight. **(N, O)** After 6 h-fasting at ZT1, CORT20 did not affect plasma clearance of [^3^H]oleate. **(P)** Upon an oral olive oil bolus at ZT1, CORT20 did not affect peak plasma triglyceride levels. **(Q)** DEX and CORT20 similarly induced *Fkbp5* expression in iBAT, sWAT, gWAT and liver. In hippocampus, *Fkbp5* expression was increased by CORT20, and decreased by DEX. **(R)** *Mt2a* expression was similarly induced by DEX and CORT20 in iBAT, sWAT, gWAT, liver and hippocampus. Statistical significance was calculated using 2-way ANOVA analysis in **(A, I)**; with one-way ANOVA analysis in **B–C, G–H, J–M, O, Q-S** and with linear mixed models in D-F, N and P. Depicted below the graphs are the P-values of the ANOVA tests for either steroid treatment (S), Time (T) or the interaction between treatment and time **(I)**. *P < 0.05, ** P < 0.01, ***P < 0.001. 'ns' means 'not significant'.

To exclude that differences between dexamethasone and low-dose corticosterone treatments were due to differences in effective dose and not to differential MR/GR affinities, we investigated mice that received a higher dosage of corticosterone (20 mg rather than 5 mg), and compared these to vehicle- and dexamethasone-treated animals. High-dose corticosterone strongly elevated plasma corticosterone levels and reduced adrenal weight but not spleen weight (**Figures 6A–C**). High-dose corticosterone induced a rapid drop of body weight during the first week of treatment, due to a reduction of lean mass, which normalized during the rest of the experiment (**Figures 6D, E**). In contrast to the fat mass loss after dexamethasone, high-dose corticosterone increased total fat mass (**Figure 6F**). Plasma glucose levels were not influenced by high-dose corticosterone, while it induced insulin levels stronger than dexamethasone (**Figures 6G, H**). High-dose corticosterone did not affect plasma glucose clearance nor peripheral glucose uptake, plasma free fatty acid levels, plasma triglycerides, liver weight, uptake of triglyceride-derived fatty acids in peripheral tissues or postprandial triglyceride levels (**Figures 6I–P**). To estimate corticosteroid receptor activation, *Fkbp5, Gilz* and *Mt2a* expression was measured in iBAT, sWAT, gWAT, liver and hippocampus (**Figures 6Q, R**). High-dose corticosterone and dexamethasone similarly induced *Fkbp5* expression in all peripheral tissues (**Figure 6Q**). In the hippocampus, *Fkbp5* expression was increased by high-dose corticosterone, but decreased by dexamethasone (**Figure 6Q**). In iBAT, sWAT, gWAT, liver and hippocampus, *Mt2a* expression was similarly induced by high-dose corticosterone and dexamethasone (**Figure 6R**). In conclusion, effects of high-dose corticosterone were different from those of dexamethasone.

### Aldosterone add-on therapy does not influence metabolic effects of dexamethasone

**Figure 7:**
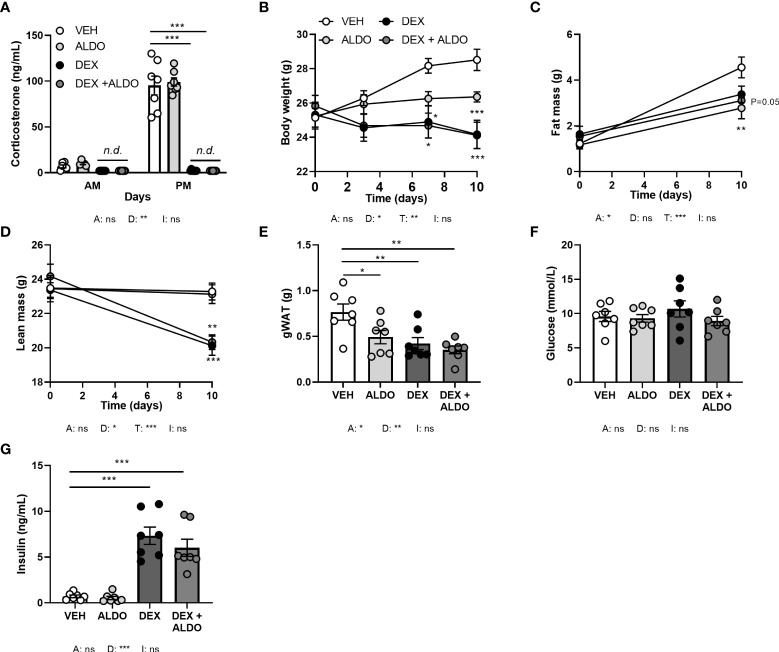
Aldosterone add-on treatment did not influence the metabolic effects of dexamethasone. Mice received a high-fat diet mixed with dexamethasone (DEX) or vehicle (VEH) and were subcutaneously implanted with aldosterone (ALDO) or vehicle pellets for 10 days. **(A)** ALDO did not affect corticosterone levels at AM (ZT1) and PM (ZT11) of the light phase, while CORT levels were not detected (n.d.) after DEX and DEX + ALDO treatment. **(B–D)** DEX + ALDO did not influence the DEX-induced reduction of body weight, fat mass and lean mass. **(E, F)** 6 h-fasted plasma glucose or insulin levels were unaffected by ALDO and DEX + ALDO. **(G)** ALDO, DEX and DEX + ALDO reduced gonadal white adipose tissue (gWAT) weight Statistical significance was calculated using 3-way ANOVA in (A-D) and 2-way ANOVA analysis in **(E–G)**. Depicted below the graphs are the P-values of the ANOVA tests for either ALDO (A), DEX (D), or the interaction between ALDO and DEX (I). *P < 0.05, ** P < 0.01, ***P < 0.001. 'ns' means 'not significant'.

To investigate whether MR contributes to the effects of corticosterone add-on treatment, we evaluated the effect of dexamethasone treatment combined with MR-specific agonist aldosterone, under the assumption that high aldosterone can occupy receptors no longer activated upon corticosterone depletion. Mice received aldosterone (0.3 mg) or vehicle pellets in combination with a HFD with or without added dexamethasone for 10 days. Plasma aldosterone levels likely were in a supraphysiological range, as a pilot study showed strongly elevated plasma aldosterone levels compared to control mice (data not shown). Dexamethasone again strongly diminished corticosterone levels, while aldosterone did not influence them (**Figure 7A**). Aldosterone treatment decreased fat mass gain (main effect: P<0.05), and did not influence body weight or lean mass (**Figures 7B–D**). In line with the reductions in total fat mass, aldosterone and dexamethasone independently reduced gWAT weight (**Figure 7E**). Glucose levels were unaffected by both aldosterone and dexamethasone treatment (**Figure 7F**), and aldosterone did not influence insulin levels, while dexamethasone caused hyperinsulinemia (**Figure 7G**). Aldosterone alone or as add-on treatment to dexamethasone did not affect other metabolic parameters, such as plasma triglycerides, free fatty acid levels, and tissue weights of liver and iBAT (data not shown). All in all, aldosterone-on did not prevent nor exacerbate dexamethasone effects and thus differed from corticosterone add-on.

### MR antagonist eplerenone reduces the glucose intolerance caused by corticosterone add-on

**Figure 8:**
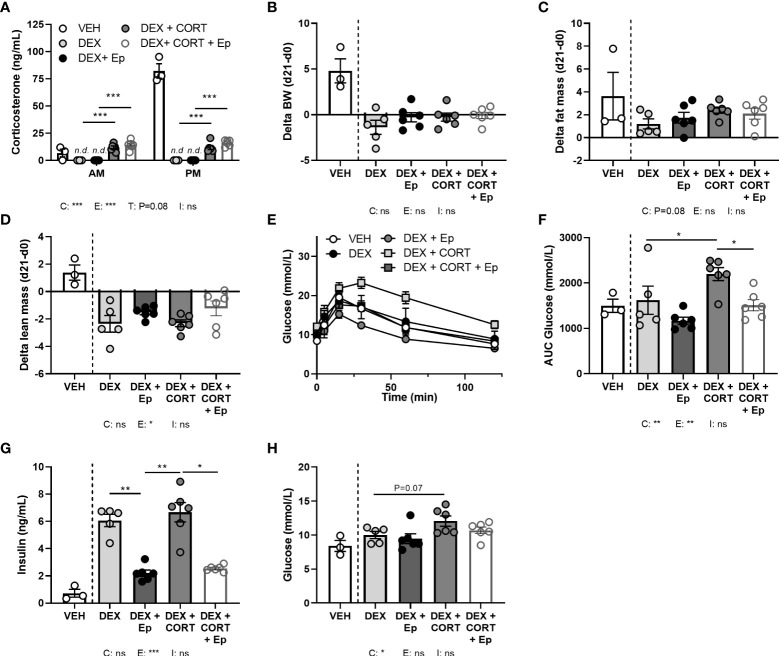
Eplerenone reduces the glucose intolerance and hyperinsulinemia caused by corticosterone add-on. Mice received a high-fat diet mixed with vehicle (VEH), dexamethasone (DEX) with or without eplerenone (Ep) and were subcutaneously implanted with corticosterone (CORT) or vehicle pellets for 3.5 weeks. **(A)** After Ep, plasma CORT levels were unaltered at AM (ZT1) and PM (ZT11) of the light phase at day 21. **(B–D)** Comparing gains per mouse (delta), Ep did not influence body weight or fat mass, but decreased lean mass. **(E, F)** DEX+CORT exacerbated oral glucose intolerance compared to DEX at ZT1 at day 10, which was prevented by Ep. **(G, H)** Ep reduced insulin levels, but not glucose levels. The vehicle group was excluded from statistical analyses. Statistical significance was calculated using 2-way ANOVA analysis in **(B–D** and **F–H)** and with 3-way analysis in **(A)**. Depicted below the graphs are the P-values of the ANOVA tests for either CORT (C), Ep (E) or the interaction between CORT and Ep (I). *P < 0.05, ** P < 0.01, ***P < 0.001. ‘ns’ means ‘not significant’.

**Figure 9:**
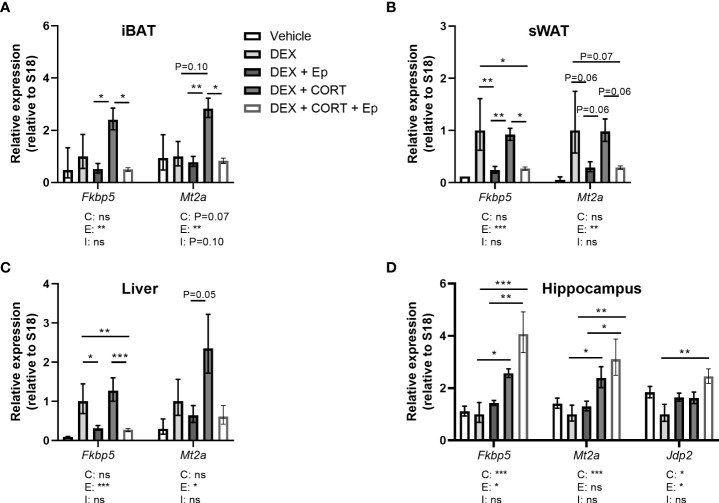
Eplerenone prevents additional induction by corticosterone add-on on target gene expression. Mice received a high-fat diet mixed with vehicle (VEH), dexamethasone (DEX) with or without eplerenone (Ep) and were subcutaneously implanted with corticosterone (CORT) or vehicle pellets for 3.5 weeks. **(A–C)** Ep reduced the induction of *Mt2a* and *Fkbp5* expression by DEX and DEX + CORT in interscapular brown adipose tissue (iBAT), gonadal white adipose tissue (gWAT) and liver. **(D)** *Fkbp5, Mt2a* and *Jdp2* expression was increased by DEX + CORT, and more so by DEX + CORT + Ep in the hippocampus. The vehicle group was excluded from statistical analyses. Statistical significance was calculated using 2-way ANOVA analysis. Depicted below the graphs are the P-values of the ANOVA tests for either CORT (C), Ep (E) or the interaction between CORT and Ep (I). *P < 0.05, ** P < 0.01, ***P < 0.001. 'ns' means 'not significant'.

In an alternative approach to investigate the role of MR in corticosterone add-on treatment, male mice received the MR antagonist eplerenone next to dexamethasone and corticosterone add-on treatment. We did not find evidence for off-target effects of eplerenone, as corticosterone plasma levels were unaltered (**Figure 8A**) and androgen-responsive seminal vesicle weight was unchanged (**Supplementary Figure S3A**). Dexamethasone again fully suppressed endogenous plasma corticosterone levels (**Figure 8A**), while corticosterone add-on increased plasma corticosterone levels (**Figure 8A**), although the levels were lower than in the first experiment (**Figure 1A**). While corticosterone add-on did not affect fat mass gain in this experiment, eplerenone did not influence fat mass gain, body weight, adipose tissue weights and adipocyte sizes (**Figures 8B, C**, **Supplementary Figures S3B–E**). Eplerenone partially prevented the reduction of lean mass caused by dexamethasone (main effect: P<0.05, **Figure 8D**). Given corticosterone add-on exacerbated dexamethasone-induced hyperglycemia and hyperinsulinemia, we evaluated oral glucose tolerance. Corticosterone add-on aggravated glucose intolerance compared to dexamethasone alone, which was prevented by eplerenone (**Figures 8E, F**). Eplerenone did not influence plasma glucose levels, but reduced the hyperinsulinemia caused by dexamethasone alone and by corticosterone add-on (**Figures 8G, H**). Eplerenone reduced brown adipose tissue weights and lipid droplet size (main effects: P<0.05) and prevented the suppression of white blood cell counts by dexamethasone (main effect: P<0.05, **Supplementary Figures S3F–J**). Basal fasted triglyceride, cholesterol and free fatty acid levels were unaffected by eplerenone (**Supplementary Figures S3K–M**). Interestingly, eplerenone fully prevented the induction of *Fkbp5* and *Mt2a* expression by corticosterone add-on in iBAT, sWAT and liver (**Figures 9A–C**), and of dexamethasone alone in sWAT and, for *Fkbp5*, in liver (**Figures 9B, C**). In the hippocampus, eplerenone induced expression of *Fkbp5*, and for MR-putative target gene *Jdp2* in the corticosterone add-on group only (main effect: P<0.05, **Figure 9D**). All in all, eplerenone prevented the exacerbated glucose intolerance and the induction of some GR/MR target genes caused by corticosterone add-on, suggesting that these effects were MR mediated.

## Discussion

Synthetic GCs are widely used in the clinic and often present with metabolic side effects (3, 34). Although it is widely assumed that these side effects are caused by hyperactivation of the GR, an alternative and non-mutually exclusive mode of action is hypoactivation of the MR. In this study we investigated the metabolic effects of corticosterone add-on treatment during dexamethasone treatment, in an attempt to dissect a possible role of MR hypoactivation. We show that corticosterone add-on treatment attenuated some, and aggravated other metabolic effects of dexamethasone. Dexamethasone reduced fat mass, likely in part mediated *via* enhanced WAT intracellular lipolysis and increased BAT metabolic activity, while corticosterone add-on treatment prevented both. In sharp contrast, corticosterone add-on treatment exacerbated the dexamethasone-induced hyperglycemia, hyperinsulinemia and glucose intolerance. The increased fat mass and aggravated hyperinsulinemia after corticosterone add-on treatment are in line with known metabolic effects of MR activation (35–41). Although some studies reported no effects of adipose-specific deletion of MR on body weight and body composition (42, 43), MR overexpression and aldosterone treatment have been shown to increase fat mass and to induce hyperinsulinemia and hyperglycemia in mice (35–37). Conversely, blockade of MR activity by MR antagonists, aldosterone synthase deletion or adipose-specific MR deletion, was shown to improve glucose tolerance and to reduce fat mass in humans and rodents (36–41). Our data are partially in line with this, as eplerenone reduced the glucose intolerance caused by corticosterone add-on. However, it remains unclear whether eplerenone can prevent the increase in fat mass by corticosterone add-on as we were unable to replicate this finding, which is likely due to the lower corticosterone levels in the corticosterone add-on group in this experiment. In BAT, MR activation has been shown to inhibit thermogenic capacity and stimulates ‘whitening’ of BAT (44, 45), which is in line with our findings that corticosterone add-on treatment counteracts the increased BAT activity upon dexamethasone treatment, while eplerenone seemed to increase BAT activity.

Corticosterone add-on treatment modulated the dexamethasone-induced expression of several glucocorticoid target genes (e.g. *Fkbp5, Mt2a, Atgl*). We speculate that this additional effect is due to MR activation (46), but have no direct evidence for this notion at the cellular level. We cannot fully exclude that corticosterone add-on treatment enhanced dexamethasone-induced *Fkbp5* and *Mt2a* expression *via* additional GR activation, although this is unlikely given the 25-fold higher potency of dexamethasone relative to corticosterone (47) and our observation that eplerenone fully prevented the additional increase mediated by corticosterone add-on. Because eplerenone decreased the *Fkbp5* induction and hyperinsulinemia caused by dexamethasone alone, our data also suggest that dexamethasone at the given dose can act through MR, which is also in line with previous observations (17). Additional effects of MR activation on top of GR activation involve the binding to common GR response elements (GREs) in target genes, based on the very similar DNA binding domain of MR and GR. The *Fkbp5* GRE-containing regulatory region is bound by both MR and GR *in vivo* (30, 48, 49), and the apparent additive effects of MR and GR may therefore involve sequential binding of homodimers, heterodimers or even higher order mixed MR/GR complexes (50, 51). Expression of genes like *Atgl* that show reversal of effects of dexamethasone after corticosterone may contain separate GR and MR sensitive regulatory regions, or have GREs where the receptors functionally compete. Indeed, different configurations of corticosteroid receptor complexes can enhance (52) or inhibit the homodimer-mediated transcriptional response (53, 54).

Against our expectations, we found that the metabolic effects of aldosterone add-on treatment minimally affected dexamethasone effects. In our adrenally intact mice endogenous aldosterone was not depleted in the control group, but concentrations of plasma/tissue hormone levels of aldosterone-treated mice likely were in a supraphysiological range, likely resulting in overactivation of the receptors. Possibly, the aldosterone-MR complex may have induced other downstream pathways compared to the corticosterone-MR complex, by recruiting a distinct transcriptional complex (55). However, it is hard to predict what happens with occupancy of MRs in our experimental settings as corticosterone may still compete with aldosterone in cells not expressing 11βHSD type 2. Given the opposite effects of aldosterone on fat mass between our study and a previous study in adrenalectomized animals, it is likely that MR occupancy in intact animals is suboptimal upon additional aldosterone administration (56). The differences between aldosterone and corticosterone add-on treatment may also be explained by the different pharmacokinetic properties of aldosterone compared to corticosterone [e.g. poor brain penetrance (57)] or hemodynamic changes induced by aldosterone that influence the metabolic response (58). Given that eplerenone inhibited the effects of corticosterone add-on treatment on e.g. glucose intolerance, it is nevertheless likely that the corticosterone add-on effects were mediated *via* MR.

The strong reduction of fat mass after dexamethasone seems to be mediated by a redistribution of lipids from WAT towards BAT. In BAT, the lipids were most likely utilized for thermogenesis, although we did not directly measure this. Elevated FFA release from WAT *via* increased ATGL activity seems crucial in this process, especially because *Atgl* is a known GR target gene (59). FFAs also stimulate pancreatic insulin release and could thereby contribute to the insulin resistance we observed in our study (60–62). Corticosterone add-on treatment prevented the lipid redistribution that was observed upon dexamethasone treatment, by reducing both WAT lipolysis and BAT activity. Corticosterone add-on treatment aggravated hyperinsulinemia, hyperglycaemia and glucose intolerance, which may be attributed to increased hepatic gluconeogenesis, hepatic steatosis or to direct effects on the pancreas itself (63). Despite the high insulin levels and presumed insulin resistance, corticosterone add-on treatment increased the uptake of glucose in WAT and BAT. This may point towards insulin-independent regulation of GLUT4 transporters by GC, as has previously been observed in cultured brown adipocytes treated with dexamethasone (64).

We found that dexamethasone stimulated ‘futile cycling’ of nutrients, especially in the WAT, where it simultaneously increased the uptake and release of triglyceride-derived fatty acids. This is a well-established effect of GCs: by simultaneously promoting catabolic and anabolic pathways, the overall flux of lipids and glucose is increased which promotes metabolic flexibility during stress, and replenishes nutrient stores during stress recovery (65). However, we found that the overall effect of dexamethasone on fat metabolism was catabolic, given the loss of fat mass, while effects of corticosterone were more anabolic, given the increased fat mass. This disparity between dexamethasone and corticosterone may be driven by a differential balance between GR and MR activation, as in full-body GR and MR knock-out zebrafish the MR was found to be essential for triglyceride accumulation, while GR was required for catabolic processes (66).

To conclude, this study shows that metabolic effects of dexamethasone are in part mediated *via* suppression of endogenous corticosterone. Under dexamethasone treatment, the effects of corticosterone add-on treatment were similar to known metabolic effects of MR and could partially be reversed by eplerenone. To reduce metabolic side effects, in addition to psychological side effects, of synthetic GC treatment, we propose that MR should be fully (re-)activated in the brain, while in the periphery restoration of only part of MR functionality is beneficial.

## Data availability statement

The raw data supporting the conclusions of this article will be made available by the authors, without undue reservation.

## Ethics statement

The animal study was reviewed and approved by the Instantie voor Dierenwelzijn, at Leiden University Medical Center.

## Author contributions

LK, JK and OM conceived the set-up and wrote the paper. MM, ES-L and MG conceived and performed the LC-MS analysis. LK, JK, SK, JS, MV, AN planned and executed the animal experiments and subsequent analyses. All authors discussed the analyses, and gave critical input on the manuscript.

## Funding

JK was funded by Corcept Therapeutics support to OM, and LK by a grant from the Board of Directors of Leiden University Medical Center.

## Acknowledgments

The authors gratefully thank Trea C.M. Streefland, Amanda C.M. Pronck and Niek Blomberg for their valuable technical assistance.

## Conflict of interest

The authors declare that the research was conducted in the absence of any commercial or financial relationships that could be construed as a potential conflict of interest.

## Publisher’s note

All claims expressed in this article are solely those of the authors and do not necessarily represent those of their affiliated organizations, or those of the publisher, the editors and the reviewers. Any product that may be evaluated in this article, or claim that may be made by its manufacturer, is not guaranteed or endorsed by the publisher.
