# Supplementary material for: Dexamethasone-associated metabolic effects in male mice are partially caused by depletion of endogenous corticosterone
Source: Front Endocrinol (Lausanne). 2022 Aug 10;13:960279. doi: 10.3389/fendo.2022.960279 (PMC9399852; doi:10.3389/fendo.2022.960279)
Supplement: Supplementary file 1 [file DataSheet_1.docx]

SUPPLEMENTARY INFORMATION

Title: Dexamethasone-associated metabolic effects in male mice are partially caused by depletion of endogenous corticosterone

**Lisa L. Koorneef^1,2^,** **Merel van der Meulen^1,2^, Sander Kooijman^1,2^, Elena Sánchez-López^3^, Jari F. Scheerstra^1,2^, Maaike C. Voorhoeve^1,2^, Ajith N. Nadamuni Ramesh^1,2^, Patrick C. N. Rensen^1,2^, Martin Giera^3^, Jan Kroon^1,2^**^†^**, Onno C. Meijer^1,2^** ^†^**^*^**

^1^Department of Internal Medicine, Division of Endocrinology, Leiden University Medical Center, Leiden, The Netherlands;

^2^Einthoven Laboratory for Experimental Vascular Medicine, Leiden University Medical Center, Leiden, The Netherlands;

^3^Center for Proteomics & Metabolomics, Leiden University Medical Center, Leiden, The Netherlands.


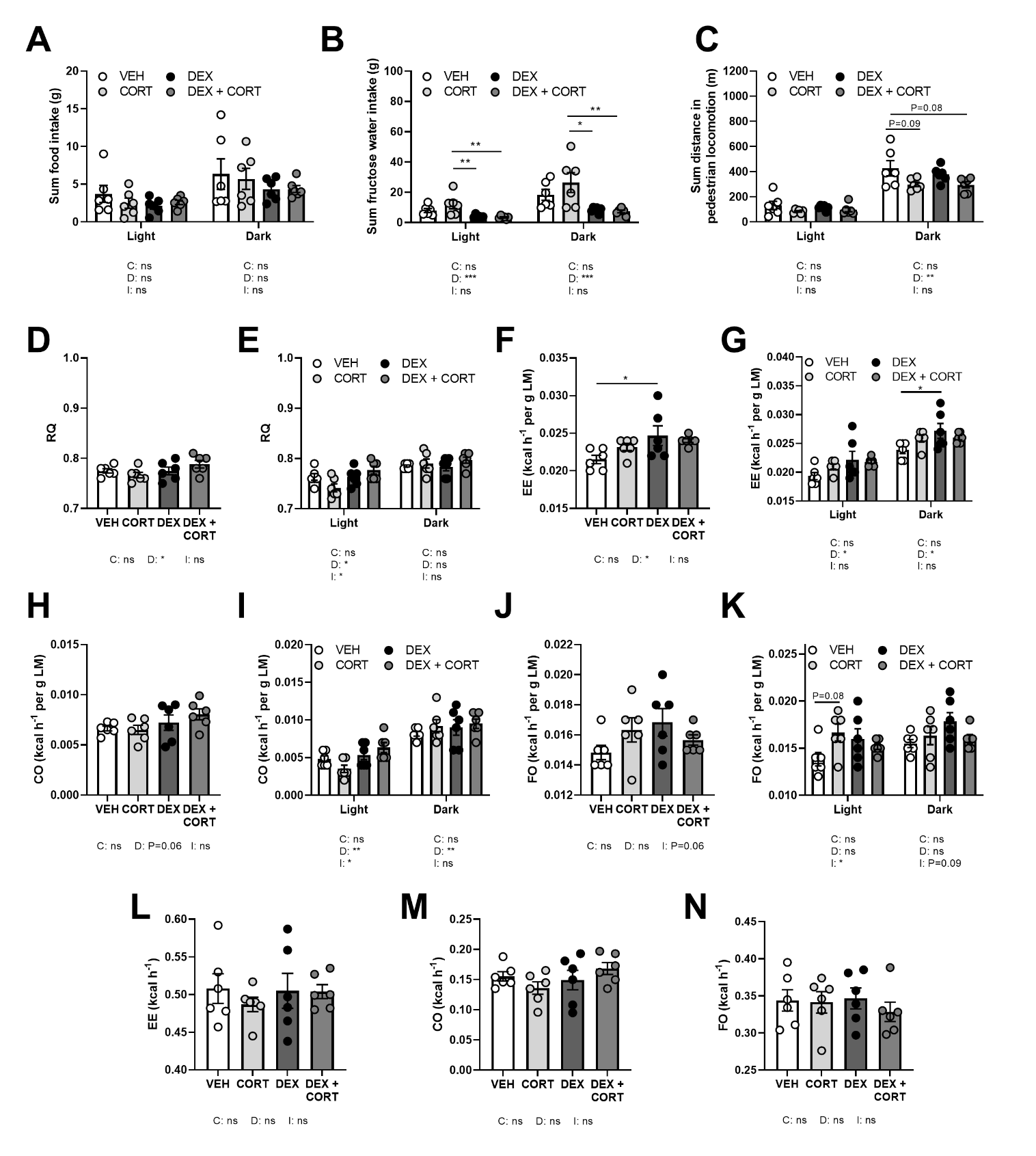
SUPPLEMENTARY FIGURES

**Supplementary Figure 1: Dexamethasone treatment increases energy expenditure.** Mice received a high-fat diet mixed with dexamethasone (DEX) or vehicle (VEH) and were subcutaneously implanted with corticosterone (CORT) or vehicle pellets for 3.5 weeks. At day 0-7, mice were housed in metabolic cages. Results are the sum of a 72 h measurement taken between day 3 and 5. (A-C) DEX reduced fructose water intake and locomotor activity, but did not affect food intake. (D-G) DEX increased the light and total respiratory quotient (RQ) and energy expenditure (EE). (H-I) DEX increased carbohydrate oxidation (CO) during the light phase. (J-K) CORT tended to increase fat oxidation (FO) in the light phase. (L-N) Effects on EE, CO and DO only occurred after the lean mass correction. Statistical significance was calculated using 2-way ANOVA analysis in each light phase (Light: ZT0 - ZT12; Dark: ZT12 - ZT24) separately. Depicted below the graphs are the P-values of the ANOVA tests for either CORT (C), DEX (D) or the interaction between CORT and DEX (I). *P < 0.05, ** P < 0.01, ***P < 0.001.

**
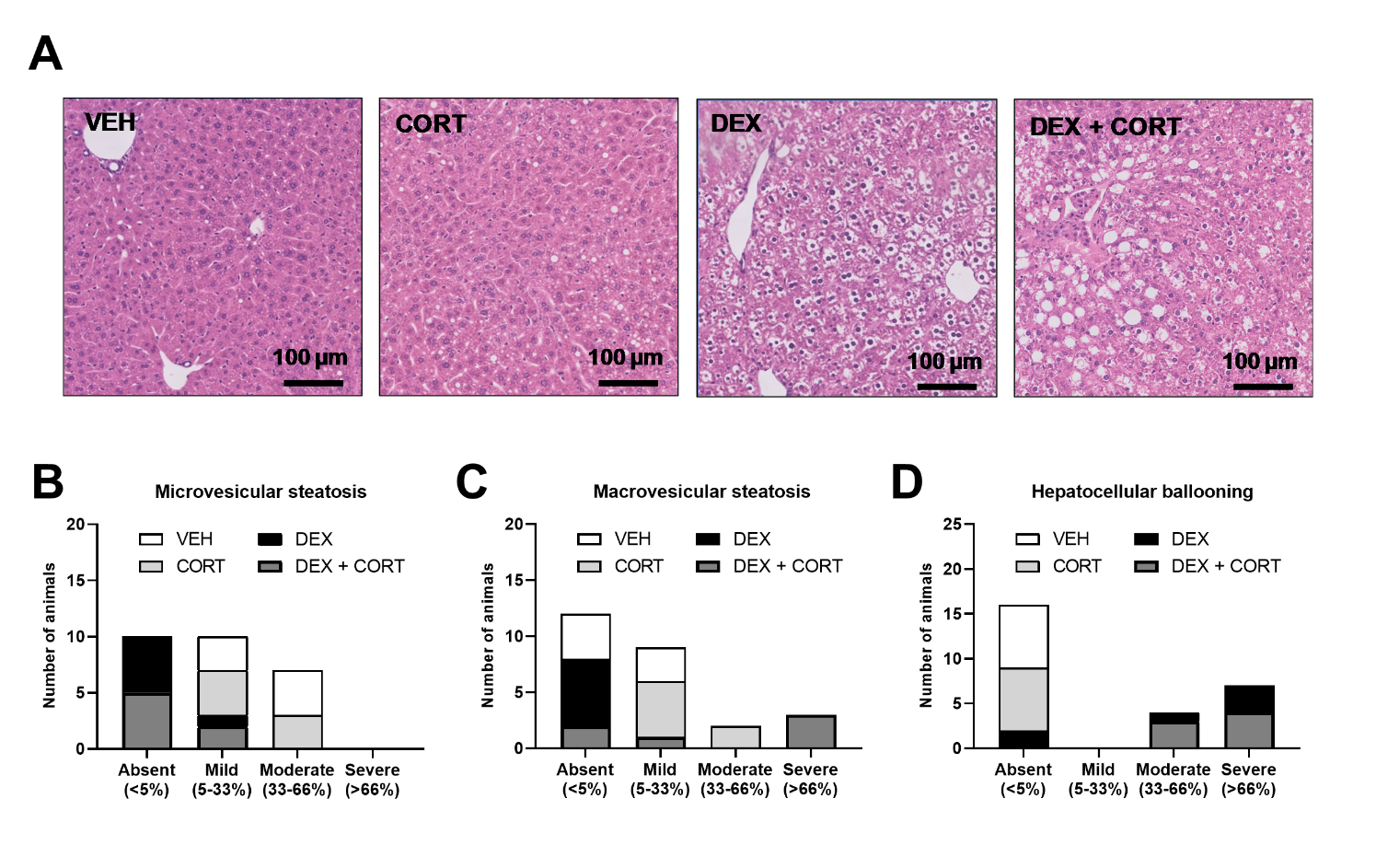
Supplementary Figure 2: Corticosterone causes hepatic macrosteatosis, while dexamethasone causes hepatocellular ballooning.** Mice received a high-fat diet mixed with dexamethasone (DEX) or vehicle (VEH) and were subcutaneously implanted with corticosterone (CORT) or vehicle pellets for 3.5 weeks. (A) Livers were examined by H&E-staining. (B) A mild degree of microvesicular steatosis was observed in VEH-treated mice, a mild-to-moderate degree in CORT-treated mice. (C) CORT and DEX + CORT induced macrovesicular steatosis. (D) Hepatocellular ballooning was observed in both DEX and DEX + CORT treatment groups.


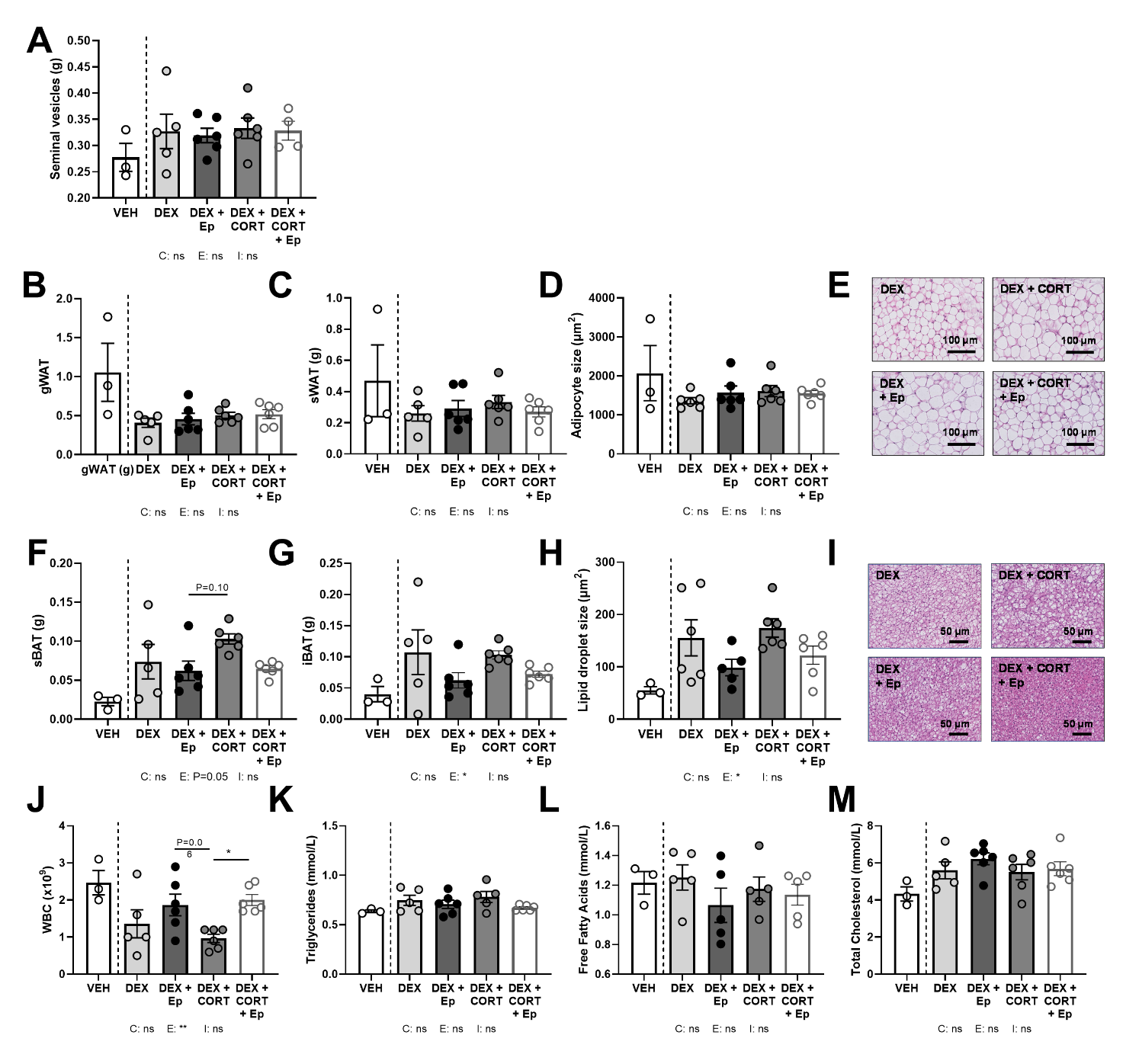
**Supplementary Figure 3: Eplerenone reduces brown fat weight and intracellular lipid droplet size.** Mice received a high-fat diet mixed with vehicle (VEH), dexamethasone (DEX) with or without eplerenone (Ep) and were subcutaneously implanted with corticosterone (CORT) or vehicle pellets for 3.5 weeks. (A) Ep did not affect seminal vesicle weight. (B-E) None of the steroid treatments affected gonadal white adipose tissue weights (gWAT) or adipocyte size as measured by H&E staining. (F-I) Ep reduced suprascapular and interscapular brown adipose tissue weight (sBAT and iBAT, respectively) and reduced lipid droplet size in iBAT. (J) White blood cell count was reduced by Ep. (K-M) None of the steroid treatment affected 6 h-fasted plasma triglyceride, free fatty acid and total cholesterol levels collected at ZT1 at day 10. The vehicle group was excluded from statistical analyses. Statistical significance was calculated using 2-way ANOVA analysis. Depicted below the graphs are the P-values of the ANOVA tests for either CORT (C), Ep (E) or the interaction between CORT and Ep (I). *P < 0.05.

SUPPLEMENTARY TABLES

**Supplementary Table 1: The multiple reaction monitoring (MRM) transitions that were used for identification of the different steroids.**

| **Steroid** | **Nominal mass (Da)** | **[M+H]+ (m/z)** | **Experimental MRM transition (Q1 -> Q3, m/z)** | **Declustering potential (V)** | **Collision energy (V)** | **Collision cell exit potential (V)** |
| --- | --- | --- | --- | --- | --- | --- |
| Cortisol | 362 | 363.2 | 363.3 -> 121.1 | 66 | 31 | 14 |
| Cortisone | 360 | 361.2 | 361.1 -> 163.1 | 111 | 31 | 10 |
| Corticosterone | 346 | 347.2 | 347.1 -> 91.0 | 56 | 77 | 10 |
| 11-dehydrocorticosterone | 344 | 345.2 | 345.1 -> 121.1 | 76 | 33 | 14 |
| Dexamethasone | 392 | 393.2 | 393.0 -> 355.2 | 26 | 15 | 18 |
| Testosterone | 288 | 289.2 | 289.1 -> 97.0 | 81 | 27 | 10 |
| Dihydrotestosterone | 290 | 291.2 | 291.1 -> 255.1 | 61 | 23 | 14 |
| Progesterone | 314 | 315.2 | 315.1 -> 109.0 | 66 | 31 | 10 |
| Cortisol-d4 (IS) | 366 | 367.2 | 367.1 -> 121.1 | 186 | 31 | 14 |
| Testosterone-d3 (IS) | 291 | 292.2 | 292.1 -> 97.0 | 106 | 29 | 10 |
| Progesterone-d9 (IS) | 323 | 324.2 | 324.1 -> 100.0 | 81 | 29 | 12 |

**Supplementary Table 2: Primer sequences that were used for RT-qPCR analysis**

| **Gene** | **Primer fw** | **Primer rev** |
| --- | --- | --- |
| *aP2* | ACACCGAGATTTCCTTCAAACTG | CCATCTAGGGTTATGATGCTCTTCA |
| *Apob* | GCCCATTGTGGACAAGTTGATC | CCAGGACTTGGAGGTCTTGGA |
| *Atgl* | ACAGTGTCCCCATTCTCAGG | TTGGTTCAGTAGGCCATTCC |
| *β2-microglobulin* | TGACCGGCTTGTATGCTATC | CAGTGTGAGCCAGGATATAG |
| *Cd36/FAT* | GCAAAGAACAGCAGCAAAATC | CAGTGAAGGCTCAAAGATGG |
| *Fkbp5* | GCCGACTGTGTGTGTAATGC | CACAATACGCACTTGGGAGA |
| *Gilz* | TGGCCCTAGACAACAAGATTGAGC | CCACCTCCTCTCTCACAGCAT |
| *Hsl* | AGACACCAGCCAACGGATAC | ATCACCCTCGAAGAAGAGCA |
| *Jdp2* | TACGCTGACATCCGCAACAT | CGTCTAGCTCACTCTTCACGG |
| *Lpl* | CCCTAAGGACCCCTGAAGAC | GGCCCGATACAACCAGTCTA |
| *Mt2a* | ACGTCCTGAGTACCTTCTCCT | GGAGGTGCACTTGCAGTTCTTG |
| *Mttp* | CTCTTGGCAGTGCTTTTTCTCT | GAGCTTGTATAGCCGCTCATT |
| *Pgc1a* | TGCTAGCGGTTCTCACAGAG | AGTGCTAAGACCGCTGCATT |
| *Prdm16* | ACTTTGGATGGGAGCAGATG | CTCCAGGCTCGATGTCCTTA |
| *Ptgds* | GCTCCTTCTGCCCAGTTTTCCT | GCCCCAGGAACTTGTCTTGTT |
| *S18* | AGGACCTGGAGAGGCTGAAG | CAGTGGTCTTGGTGTGCTGA |
| *Ucp1* | TCAGGATTGGCCTCTACGAC | TGCATTCTGACCTTCACGAC |

**Supplementary Table 3: Tissue expression of *Nr3c1* and *Nr3c2*.** Mice received a high-fat diet mixed with dexamethasone (DEX) or vehicle (VEH) and were subcutaneously implanted with low-dose (5 mg) corticosterone (CORT) or vehicle pellets for 3.5 weeks. Depicted are the mean CT-values ±SD.
